# A Low-Latency Divider Design for Embedded Processors

**DOI:** 10.3390/s22072471

**Published:** 2022-03-23

**Authors:** Xiaotong Wei, Ying Yang, Jie Chen

**Affiliations:** 1 New Technology Development Department, Institute of Microelectronics of the Chinese Academy of Sciences, Beijing 100029, China; xiaotong_wwei@163.com (X.W.); jchen@ime.ac.cn (J.C.); 2 University of Chinese Academy of Sciences, Beijing 100029, China

**Keywords:** embedded processors, divider, compound adders, shift alignment

## Abstract

Division is generally regarded as a low-frequency, high-latency operation in integer operations. Division is also the operation that stalls the processor pipeline most frequently. In order to improve the overall performance of embedded processors, a low-delay divider for embedded processors was designed. Based on the non-restoring algorithm, the divider uses a compound adder to execute addition and subtraction simultaneously and reduces the iteration path delay. By shifting the operands to align the most effective bits, the divider dynamically adjusts the number of iteration cycles to reduce the average number of cycles in the division process. The divider design was simulated by Modelsim and implemented on a FPGA board for verification. Synthesized in a Semiconductor Manufacturing International Corporation (SMIC) 65 nm Low Leakage process, the achieved frequency of the design was up to 500 MHz and the area cost was 5670.36 μm^2^. Compared with other dividers, the proposed divider design can reduce the delay of single iteration by up to 45.3%, save the average number of iteration cycles by 20–50%, and save the area by 23.3–86.1%. Compared with other dividers implemented on FPGA, it saves LUTs by 36.47–59.6% and FFs by 67–84.28%, runs 2–6.36 times faster. Therefore, the proposed design is suitable for embedded processors that require low power consumption, low resource consumption, and high performance.

## 1. Introduction

The float-point unit (FPU) is an operational accelerator integrated in modern embedded microprocessor cores based on the architecture such as ARM or MIPS. However, in general, it also requires a larger hardware cost and dynamic power consumption. The microprocessor cores in certain fields have strict limitations on power consumption and hardware cost, and may not support the floating-point operations in hardware. Therefore, such operations are implemented by fixed-point units, and this leads to a high latency system.

Division is the slowest and most complex operation among the four basic operations [1]. Although the division operation is less frequent than the two basic arithmetic operations, addition and multiplication, it significantly affects the performance of the microprocessor core. It has been shown that the division operation accounts for only 3% of all arithmetic instructions, but accounts for 40% of the processor stall delay [2]. There is a significant need to improve the fixed-point design of dividers for the overall performance of processors. Embedded processors have strict restrictions on power consumption and logic area, so an efficient and low resource consumption divider design is necessary [3].

Divider design in embedded processors uses two main types of algorithms: the first type is the digital recursive method, such as the restoring algorithm [4,5] and non-restoring [6] algorithm; the second type is the iterative approximation method, such as Newton–Raphson algorithm [7,8,9], the CORDIC algorithm [10,11,12], and the Goldschmidt algorithm [13,14,15,16]. Iterative approximation methods can be designed as a pipeline structure, and the convergence speed is faster than that of digital recursive methods. Thus, divider designs that pursue high performance are suitable for these methods. However, iterative approximation methods require multiple full-precision multipliers. Restricted by multiply units, the dividers with iterative approximation method architectures have an excessively long critical path, which limits the increase in the frequency. Moreover, multipliers occupy a large area, which is unacceptable for area-sensitive embedded processors. Compared to the iterative approximation algorithms, the digital recursive approaches have better power and area consumption efficiency. The approaches use only adders and shift registers in an iterative process, and do not need to execute multiplication. However, the convergence rate is linear with the divider bit width n, which inevitably leads to many iterative cycles and poor performance of dividers. To address the problem, the research proposed in this paper proposes the shift alignment strategy. Therefore, digital recursion methods are the first choice for embedded processors to implement divider design [17]. 

The restoring algorithm [18,19] first shifts the divisor left so that the least significant bit (LSB) is aligned with the most significant bit (MSB) of the dividend. In the iterative process, the operands’ size is compared, subtraction and addition are optionally preformed to recover the partial remainder, and the divisor is shifted right by 1 bit. It then loops to repeat the above operations. Its advantage lies in simple control logic, but the drawbacks are high computational delay, large area consumption, and the number of iteration cycles. The non-restoring algorithm [19,20,21,22] adds a certain control logic to the restoring algorithm. Only one subtraction or addition is performed per iteration, and no additional restoring operations are required. The non-restoring algorithm chooses to perform addition or subtraction according to the quotient digit, which is selected from {−1, 1}. Compared with the restoring algorithm, the iteration delay of the non-restoring algorithm is lower, but the number of iteration cycles is not reduced, and it still uses the dividend bit width. Compared with the non-restoring algorithm, the Radix-2 SRT algorithm introduces 0 into the quotient set, which is {−1, 0, 1}, and the iterative operation only needs to shift the operands in some judgments, which reduces the average delay of the division. Radix-2 SRT is actually similar to the non-restoring algorithm. However, its quotient selection logic is more complex. Most embedded processor floating-point units use the SRT algorithm [2,21,23,24,25]. The SRT algorithm has different iteration cycles depending on the radix. The radix of the SRT algorithm is selected as an integer power of 2. The larger the radix, the more quotient digits will be returned in each iteration, and the number of iteration cycles is lower. However, this will lead to more complex quotient selection logic, longer quotient selection key paths, and more hardware resource consumption [26,27]. Therefore, for the design of the divider in embedded processors, in order to meet the resource restriction requirements, a radix of 2 is generally adopted. This also means that the high-performance Radix-4 SRT algorithm and algorithms with higher radix are difficult to apply here.

Aimed at addressing the conflict between the performance and resource consumption, the divider design proposed in this paper based on the non-restoring algorithm uses a compound adder to perform addition and subtraction operations at the same time, which can further reduce the iterative path delay. Moreover, it changes the strategy of shifting operands to reduce the number of iteration cycles. At the same time, the design consumes less hardware resources.

The rest of the paper is organized as follows. In Section 2, the theoretical analysis of the proposed algorithm is presented. Section 3 describes the structure of the proposed divider. The implementation results and discussion with other divider designs are provided in Section 4. Conclusions are then briefly drawn in Section 5.

## 2. Algorithm Analysis

The proposed divider design, based on the non-restoring algorithm, performs the addition and subtraction at the same time during each iteration to further increase the operation frequency; by normalizing the most effective bit position of the dividend and the divisor, the number of iteration cycles is reduced.

### 2.1. Reduce Path Delay

The restoring algorithm is similar to manual operation. The divisor is initialized to shift left by (*n*−1) bits, and *n* is the bit width of the dividend, so that the LSB of divisor is aligned with the MSB of the dividend. The divisor is subtracted from the partial remainder in each iteration process.

(1) z′[i+1]=z[i]−d[i],

In Formula (1), *z*[*i*] is the partial remainder in the *i*-th iteration process, *d*[*i*] is the result stored in the divisor register in the *i*-th iteration process, *i* represents the number of iterations. According to the sign of the result z’[*i* + 1], the quotient digit of the iteration is chosen and it is decided whether it is necessary to restore the partial remainder, as shown in (2) and (3).

(2) $$q[n-1-i]=\{\begin{array}{c} 1,z\prime [i+1]\geq 0\\ 0,z\prime [i+1]<0 \end{array},$$

(3) $$z[i+1]=\{\begin{array}{c} z\prime [i+1],z\prime [i+1]\geq 0\\ z\prime [i+1]+d[i],z\prime [i+1]<0 \end{array},$$

where *q*[*n*−1−i] is the (*n*−1−*i*)th bit of the quotient.

The divisor register iteration formula is:

(4) d[i+1]=d[i]>>1,

*d*[0] is initialized to be the divisor shifted left by (*n* − 1) bits, that is, to align the LSB of the divisor with the MSB of the dividend. After the *n* iteration processes, the result quotient is obtained, which has a width of *n* bits.

When *z*’[*i* + 1] is less than 0, the iteration path delay in the restoring algorithm includes two additions, since it needs to restore the partial remainder, whereas the non-restoring algorithm performs only one addition or subtraction per iteration, which calculates the partial remainder. The partial remainder iteration formulas are:

(5) $$z[i+1]=\{\begin{array}{c} z[i]-d[i],z[i]\geq 0\\ z[i]+d[i],z[i]<0 \end{array},$$

When *z*[*i*] is less than 0, *z*[*i*] should be restored to *z*[*i* − 1], which is the partial remainder in the last iteration, and then *d*[*i*] is subtracted. In fact, the result is equal to z[*i*] + d[*i*] in this case, as shown in (6):

(6) $$\begin{array}{ll} z[i+1] & =z[i-1]-d[i]\\ & =(z[i]+d[i-1])-d[i]\\ & =z[i]+2d[i]-d[i]\\ & =z[i]+d[i] \end{array},\;z[i]<0$$

According to the sign of the partial remainder *z*[*i*+1], the quotient digit in this iteration is selected as:

(7) $$q[n-1-i]=\{\begin{array}{c} 1,z[i+1]\geq 0\\ -1,z[i+1]<0 \end{array},$$

The quotient set is {−1, 1} [19,27,28].

According to *q*[*n*−*i*], Formula (5) can be rewritten as:

(8) z[i+1]=z[i]−q[n−i]⋅d[i],

Since *q_i_* ∈ {−1, 1}, it is also necessary to convert the −1 and 1 weightings to conventional binary digits at the end. All the positive and negative quotient digits are separated, and represent one positive binary number and one negative binary number, and then added to obtain the quotient *Q*. The final quotient result is calculated by:

(9) $$Q=\sum_{q_{i}=1}2^{i}q_{i}+\sum_{q_{i}=-1}2^{i}q_{i},$$

The iterative path delay consists of three parts: (1) select the quotient digit according to the partial remainder result, which is the delay of one 2:1 MUX (Multiplex); (2) choose to perform addition or subtraction according to the quotient digit generated by the previous iteration, which is the delay of one 2:1 MUX; (3) perform addition or subtraction, which is the delay of one adder.

The algorithm proposed in this paper performs addition and subtraction operations simultaneously through a compound adder, so that the iterative path delay is reduced by one 2:1 MUX. In the (*I* + 1)th (*i* > 0) iteration, the partial remainder iteration formulas are:

(10) $$z^{\prime }[i+1]=z[i]-d[i],\;$$

(11) $$z^{\prime \prime }[i+1]=z[i]+d[i],$$

(12) $$z[i+1]=q[n-i]?z^{\prime }[i]:z^{\prime \prime }[i],$$

According to *z*[*i*+1], the quotient digit in this iteration is generated as:

(13) $$q[n-1-i]=\{\begin{array}{c} 1,z[i+1]\geq 0\\ 0,z[i+1]<0, \end{array}$$

**Figure 1:**
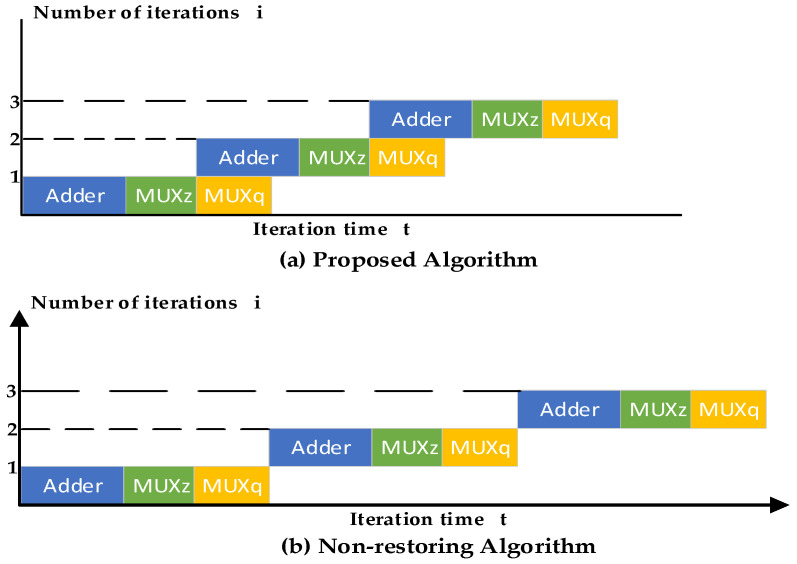
Comparison of the iterative pipeline architecture.

When the quotient selection of Formula (13) is performed, the addition or subtraction operation in the next iteration process, Formulas (10) and (11), can be started. Since the adder delay is longer than the MUX, it will not affect the choice of the partial remainder in the following operation of Formula (12). The iterative process pipeline comparison is shown in Figure 1, where Adder is the delay of addition or subtraction, MUXz is the delay of the partial remainder selection, and MUXq is the delay of the quotient digit selection. The delay time of each iteration in (a) is less than that in (b).

The quotient set of the proposed algorithm is {0, 1}, and the result quotient is directly obtained by splicing the quotient digit generated in each iteration process. The traditional non-restoring algorithm finally needs to perform a subtraction operation on the quotient set of −1 and +1 to obtain the final result [19]. Thus, the proposed algorithm also reduces the delay of one-stage adder.

### 2.2. Reduce Iteration Cycles

**Figure 2:**
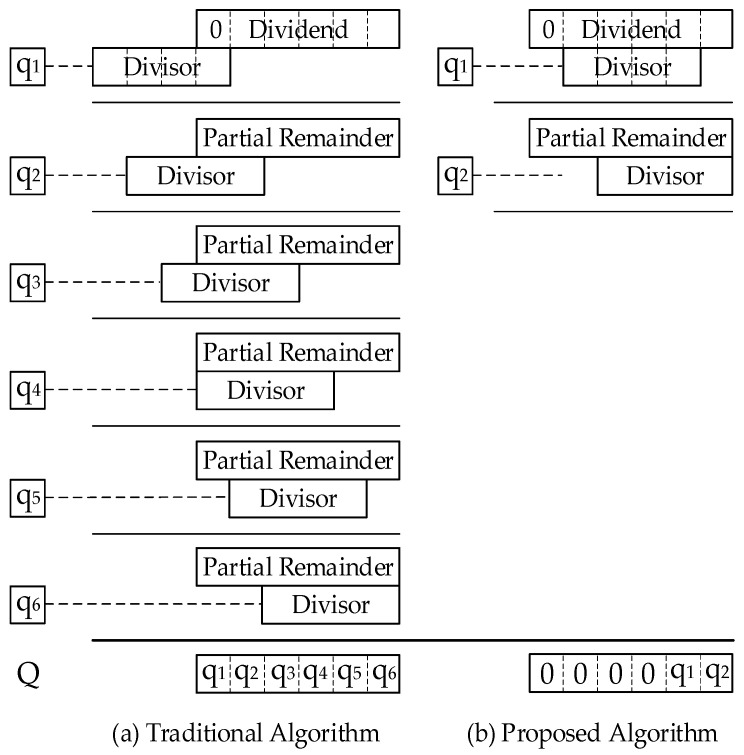
Comparison of iteration cycles.

In traditional iterative algorithms, the divisor needs to be shifted left by (*n*−1) bits for initialization. Each iteration process generates a quotient digit, and the number of iteration cycles is n, which is the dividend bit width. As shown in the left panel (a) of Figure 2, the dividend bit width n is 6 bits, and the divisor’s effective bit width is 4 bits. The divisor is shifted left by 5 bits to align the LSB of the divisor with the MSB of the dividend, for a total of 6 iterations. Since the MSB of the dividend is 0, the effective bit width is 5 bits. Until the fifth iteration, the first effective quotient digit is output. The quotient digits of the previous continuous iteration process are all 0; that is, q1, q2, q3, and q4 are 0.

In the proposed algorithm, during initialization, the most effective bits of the divisor and the dividend are aligned. The most effective bit of one data point is the most significant bit in which the digit is 1. Therefore, the quotient digit generated in the first iteration process is effective, eliminating the previous invalid iteration process and improving the efficiency of division execution. As shown in the right panel (b) of Figure 2, the most effective bits of the divisor and the dividend are normalized and aligned, and a total of two iterations are performed. In this example, the proposed algorithm saves 66.7% of the iteration cycles. For division operations in processors, the operand bit width is 32 bits. Traditional algorithms, such as the restoring, non-restoring, and Radix-2 SRT algorithms, require a fixed number of 32 iteration cycles, whereas the proposed algorithm dynamically adjusts the number of cycles from 2 to 31 according to the effective bit width of the operands, which can save more iteration cycles.

## 3. Divider Circuit Design

**Figure 3:**
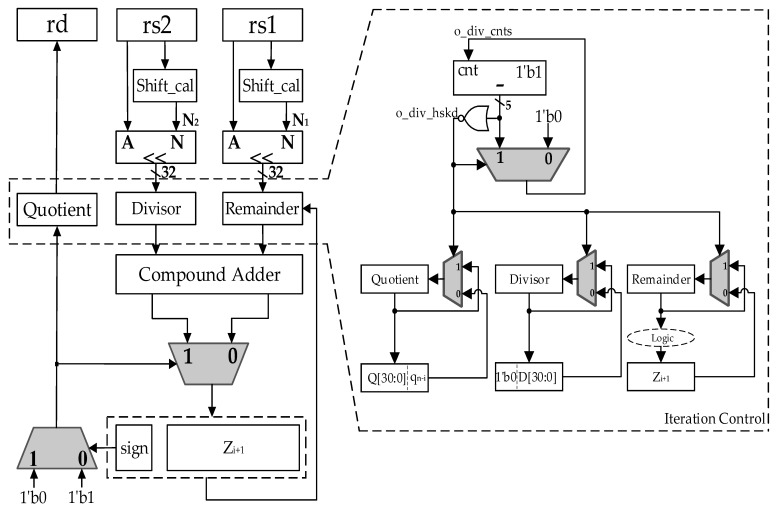
The structure of the proposed divider.

Figure 3 is the circuit architecture diagram of the algorithm proposed in this paper, taking the calculation of 32-bit division as an example. rd, rs2, and rs1 are operand registers, which store quotient, divisor, and dividend. *Z_i_*_+1_ is a partial remainder register, of which 32 bits store the result value, 1 bit stores the sign bit, and the quotient digit is selected according to the sign bit. The architecture includes three submodules, the Shift_cal module, the shift module, and the compound adder module.

The Shift_cal module checks the position of the most effective bit of the operand, and obtains the value by which the most effective bit is shifted left to the 32nd bit. The number of shift bits of the divisor is N_2_, and the number of shift bits of the dividend is N_1_, so the number of iteration cycles is (N_2_ − N_1_ + 1). If N_2_ is less than N_1_, it means that the dividend is less than the divisor. For integer division, the quotient is directly obtained as 0, and the remainder is the dividend.

Through the shift module, the most effective bits of the dividend and the divisor are normalized and aligned. The initialized dividend and divisor are stored in the remainder register and the divisor register, respectively, for iterative operations. The remainder minus the divisor and the remainder plus the divisor are performed simultaneously through the compound adder. According to the quotient digit generated in the last iteration, the addition result or the subtraction result is chosen to be passed to the partial remainder register *Z_i_*_+1_, and then the quotient digit is generated in this iteration process according to the sign bit of *Z_i_*_+1_. When the last iteration is complete, the register quotient stores the result quotient, and the partial remainder register *Z_n_* is shifted right by N_1_ bits to obtain the corrected remainder.

Control over iteration cycles enables control of the changes in the register quotient, divisor, and remainder, as shown in the dashed box. Before o_div_cnts counts to zero, which is initialized to N_2_ minus N_1_, the completion signal o_div_hskd is always zero. The register quotient, divisor, and remainder update their values on each iteration, as shown in Equations (4) and (7)–(10). Once the signal o_div_cnts reaches zero, which implies the iteration process is completed, the signal o_div_hskd is updated to 1. The register quotient, divisor, and remainder hold their values during the last iteration and do not change, and o_div_cnts remains at zero.

The iteration path goes through a 32-bit adder and a 2:1 MUX. The hardware resources used are two 32-bit adders, two 2:1 MUXs, two shift_cal modules, and two shift modules.

The details in the three submodules are described as follows.

**Figure 4:**
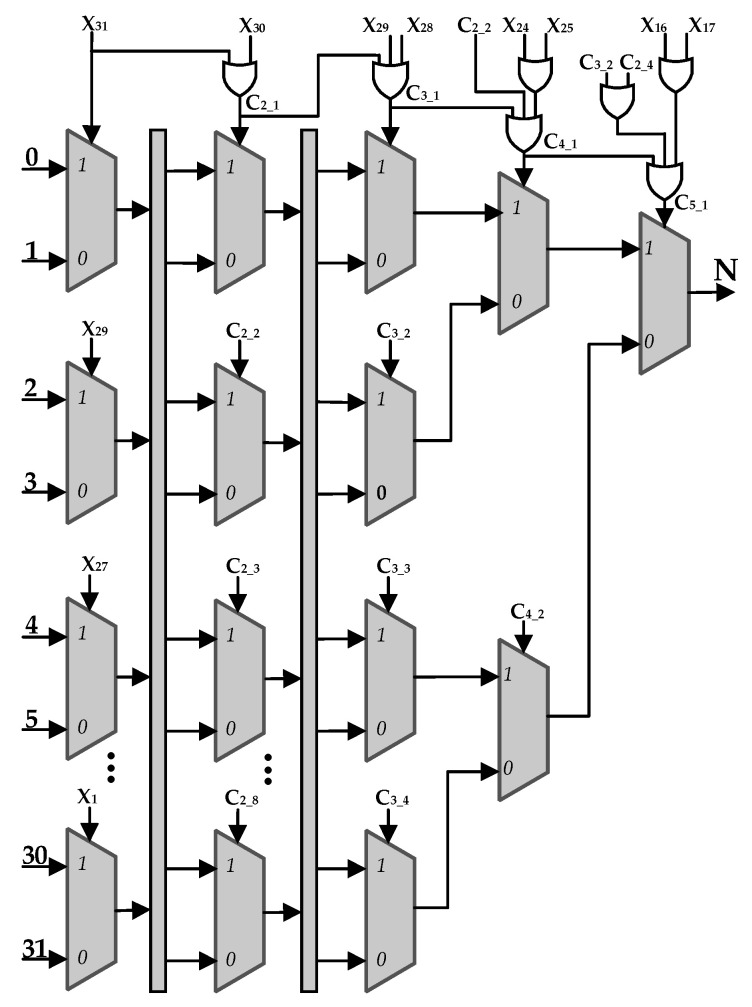
The structure of the shift_cal module.

- Shift_cal: Figure 4 shows the circuit structure of the Shift_cal module. Taking a 32-bit width input data X as an example, the module calculates the number of bits N by which X’s most effective bit is shifted left to the 32nd bit. The computational path goes through a five-stage 2:1 MUXs. The first stage has 16 MUXs, and the selection control signals are x_31_, x_29_, x_27_, …, x_1_. The inputs of the eight second-stage MUXs are the outputs of the first-stage MUXs. Similarly, the outputs of a certain level of MUXs are fed to the next level. The selection control signals for each level are listed as follows.

C_2_1_ = x_31_|x_30_,

C_2_2_ = x_27_|x_26_,

C_2_3_ = x_23_|x_22_,

......

C_3_1_ = C_2_1_|x_29_ x_28_,

C_3_2_ = C_2_3_|x_21_|x_20_,

......

**Figure 5:**
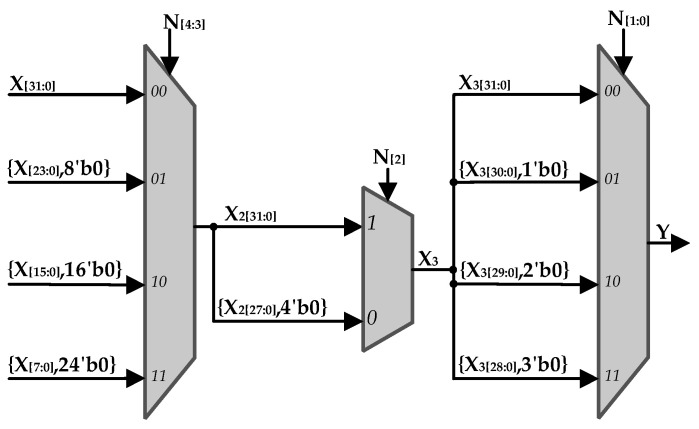
The structure of the shift module.

**Figure 6:**
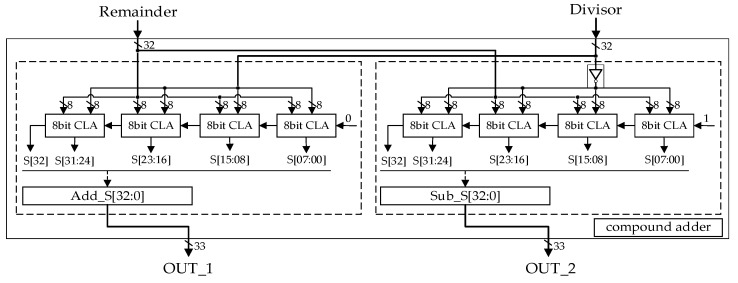
The structure of the compound adder.

C_4_1_ = C_3_1_|C_2_2_|x_25_|x_24_,

C_4_2_ = C_3_3_|C_2_6_|x_8_|x_9_,

C_5_1_ = C_4_1_|C_3_2_|C_2_4_|x_17_|x_16_,

- Shift: Figure 5 shows the structure of the shift module. According to the value of N, the input data X is shifted left by the corresponding number of bits. The weight of N_[4:3]_ is 8, so when N_[4:3]_ is 0, 1, 2, 3, X is shifted left by 0 bits, 8 bits, 16 bits, and 24 bits correspondingly. N_[2]_ has a weight of 4 and N_[1:0]_ has a weight of 1.

- Compound Adder: Figure 6 shows the architecture of the compound adder, which execute remainder plus divisor and remainder minus divisor simultaneously. It consists of two adders, each of which consists of four 8-bit sub-Carry Lookahead Adders (CLAs). The left adder calculates the result of remainder plus divisor. Remainder and divisor are spilt into four 8-bit wide sections. Every sub-CLA computes the addition result of the corresponding parts of remainder and divisor. The carry digit of the first sub-CLA is 0, and other sub-CLA’s carry digit comes from its last level sub-CLA. The right adder in Figure 6 calculates remainder minus divisor. The difference from the left is that remainder plus the complement of divisor and the carry digit of the first sub-CLA is 1. Therefore, the subtraction is converted to the addition of a two’s complement.

## 4. Results and Discussion

### 4.1. Function Simulation and FPGA Verification

**Figure 7:**
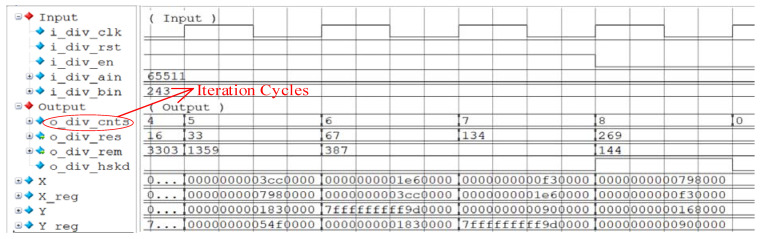
Simulation results with random numbers.

Figure 7 shows the function simulation result of the divider design using Modelsim. The dividend i_div_ain and the divisor i_div_bin are random numbers, 65,511 and 243 respectively, and the number of iteration cycles is 9. X and Y are the results of intermediate iterative operations. The counter o_div_cnts starts counting from 0. When o_div_cnts is 8, the division completion signal o_div_hskd is valid, and the final iterative results are obtained: the quotient is 269 and the remainder is 144. If the traditional non-restoring algorithm is used, the number of iteration cycles is 32. This example saves 71.8% of iteration cycles.

**Figure 8:**
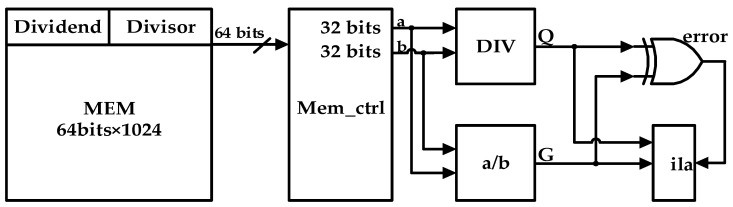
FPGA verification prototype.

The divider design was verified on the Xilinx FPGA xc7z020iclg484–1L board, and its verification prototype is shown in Figure 8.

The 32-bit dividends and divisors are stored in the storage module MEM, and these data are 32-bit random data generated by Matlab. Through the storage control module Mem_ctrl, a pair of dividend a and divisor b are read each time and passed to the subsequent operation units. The DIV module is the divider module designed in this paper, and its calculation result is the quotient Q. The a/b module is the Vivado division ip core, and its division operation obtains the standard result G. XOR is performed on the results of the two operation modules to obtain the error signal. If Q and G are different, the signal error is 1, otherwise there is no error and the signal error is always 0. During the running process, the resulting waveform is captured through the Vivado ip core ila.

**Figure 9:**
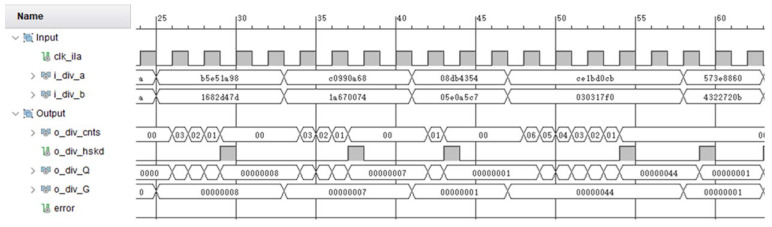
FPGA ILA capture waveform.

As shown in Figure 9, the operation result of the divider module DIV is o_div_Q, and the standard result obtained by the Vivado division ip core is o_div_G. According to different input data, the number of iteration cycles o_div_cnts changes dynamically. When each division operation of DIV is completed, that is, when o_div_hskd is 1, the results of both are compared; the error signal is always 0, and the operation result of the DIV module is correct.

### 4.2. Performance Comparison

The divider design was synthesized in the 65 nm SMIC process under the worst corner, in which the supply voltage is 1.08 V, the temperature is 125 °C, and the clock frequency is 500 MHz. The synthesis results are shown in Table 1.

Table 2 shows the delay comparison of 32-bit dividers based on different algorithms. Compared with the traditional non-restoring algorithm [28], the divider design proposed in this paper reduces the latency of a single iteration by at least 45.3%. The proposed design performs the division operation for a minimum of 2 iteration cycles and a maximum of 31, so an average of 16 iteration cycles. It reduces the average number of iteration cycles by up to 50%. Moreover, the process size used by the proposed divider design is larger. Compared with the Radix-4 SRT algorithm [29], the proposed divider reduces the average number of iteration cycles by 20%. However, a single iteration delay of the proposed divider design is larger, mainly due to the process gap.

Table 3 shows the area consumption comparison of 32-bit dividers based on different algorithms. Compared with the non-restoring algorithm [28], the divider design proposed in this paper reduces the area consumption by 86.1%. Compared with the Radix-4 SRT [29], the proposed divider design saves 23.3% of the equivalent gates.

The FPGA implementation results of the proposed divider design and other digital recursive dividers [27] are compared in Table 4. Vivado 2018.3 was used to synthesize, place, and route on the same hardware platform, which is the Xilinx Virtex UltraScale+ VCU118 board.

The proposed divider design outperforms the restoring and non-restoring dividers in both resource consumption and execution performance. In terms of resource consumption, the proposed divider design reduces LUTs by 36.47–49.5% and FFs by 82.25–84.28%. In terms of performance, the proposed divider runs 2–6.36 times faster. It performs the division operation with an average of 16 iteration cycles, and reduces the average number of iteration cycles by up to 50%.

Although Radix-2 SRT runs at a higher frequency, the division execution performance needs to be comprehensively considered in terms of frequency and the number of iteration cycles, because the execution delay of division is the product of the two. The Radix-2 SRT divider performs division for a fixed 32 cycles, whereas the proposed divider design has an average number of iteration cycles of 16. Compared with Raidx-2 SRT, the proposed divider performs division reducing the average delay by 38.87%. Moreover, the proposed divider design consumes less resources and saves 67% of FFs.

The operating frequency of the Radix-4 SRT is roughly the same as the frequency of the proposed divider, and the number of iteration cycles is fixed at 16. When the difference between the effective bit widths of the operands is less than 16, the number of iteration cycles of the proposed divider is less than that of the Radix-4 SRT. Moreover, the proposed divider design has more advantages in terms of resource consumption. Compared with Radix-4 SRT, it saves 59.6% of LUTs and 78% of FFs. This also shows that the proposed divider design has lower dynamic power consumption than Radix-4 SRT. For embedded processors that are very sensitive to resource consumption and power consumption, the divider design proposed in this paper is more suitable.

## 5. Conclusions

A low-latency divider design suitable for embedded processors is proposed. The addition and subtraction operations are performed simultaneously by the compound adder, which reduces the single iteration delay, so that the delay is only one level of adder and one level of 2:1 MUX. The number of iteration cycles can be adjusted dynamically by shifting the operands to align the most effective bits.

Based on the proposed divider architecture, a 32-bit divider circuit is implemented. According to the difference between the effective bit widths of the input operands, the proposed divider adjusts the number of iteration cycles dynamically in the range of 1–32. Compared with other digital recursive dividers, such as the restoring, non-restoring, and Radix-2 SRT dividers, it consumes fewer hardware resources, performs more efficiently, and reduces the average number of iterations by up to 50%. Compared with Radix-4 SRT, the proposed divider has better performance under limited input operands, consumes less hardware resources, and is more suitable for embedded processors.

## Figures and Tables

**Table 1:** The synthesized results of the proposed divider.

Area (μm^2^)	Number of Equivalent Gates	Dynamic Power (mA)	Critical Path Delay (ns)
5670.36	1688 ^1^	0.6116	1.9168

^1^ The equivalent gate number is obtained by dividing the total area by the area of the NAND2 gate.

**Table 2:** Comparison with other dividers in delay.

Parameters	Non-Restoring [28]	Radix-4 SRT [29]	Proposed Work
Delay (ns)	3.507	1.0	1.9168
Iteration Cycles	32	20	2–31
Total Delay (ns)	112.22	20.0	30.668 ^1^
Process (nm)	45	32	65

^1^ The total delay of the proposed work is obtained by multiplying the single delay and 16, the average iteration cycles of the proposed work.

**Table 3:** Comparison with other dividers in area.

Parameters	Non-Restoring [28]	Radix-4 SRT [29]	Proposed Work
Equivalent gates	/	2200	1688
Area (μm^2^)	40,806	1957.53	5670.36
Process (nm)	45	32	65

**Table 4:** FPGA comparison results.

Divider	Iteration Cycles		LUTs	FFs	Frequency (MHz)
	Min	Max			
Restoring	32	32	200	210	100
Non-Restoring	32	32	159	186	245
Radix-2 SRT	32	32	100	100	900
Radix-4 SRT	16	16	250	150	725
Proposed work	2	31	101	33	736

## Data Availability

Not applicable.
